# A Lively Session

**Published:** 1898-06

**Authors:** 


A Lively Session.—The thirtieth annual session of the Texas State Medical Association at Houston, April 28, 29 and 30, was enlivened by a fist fight on the rostrum. A big burly fellow from Galveston sought to whip the secretary* (as we are informed), for calling him a liar out loud in meeting, when he had

*I was not present; the only meeting I have failed to attend in sixteen years.—Ed.

"risen to a point of personal privilege" and complained, as we learn, of having been dropped from the roll of membership for non-payment of dues (in accordance with the by-laws), stating that Secretary West had treated him discourteously.

A member who had traveled six hundred miles to attend the meeting, in a personal letter to me, speaking of the disgraceful scene, says: "The meeting was a failure, the opinion of others to the contrary notwithstanding. I think such meetings do great hurt to the profession. Every one, at least during my presence, appeared disheartened and discontented, except the candidates (and there were many). I do not care to describe what I saw and heard. There were scandalous scenes enacted by Secretary West and a party by the name of Gyelts (I think that was the name).* These gentlemen passed the lie to each other; there was a collision, and the first phase of a prize fight ensued on the floor of the convention. I left the hall disgusted and amazed, and at once returned home. No civility, no decorum; everything denoting insolent rusticity—with a few bright exceptions. In fact, it seemed more of a bear garden [beer?] than a reunion of a scientific body. I forbear to say more; not for want of material, I assure you."

The JOURNAL blushes for the honor of the profession thus dragged in the dust. The JOURNAL has striven for organization of the profession on a high plane—striven for better things. We abandon the task now, and will let those in control "run the machine" to suit themselves.

*No, it wasn't; it was Gwinn, one C. L. Gwinn, of Galveston, the party who insulted and drove from his office a young lady who had made the mistake of soliciting his subscription to a journal of which her father was editor, because he had the execrable taste to not like said editor.—ED.

